# Brain structural connectome in neonates with prenatal opioid exposure

**DOI:** 10.3389/fnins.2022.952322

**Published:** 2022-09-16

**Authors:** Ramana V. Vishnubhotla, Yi Zhao, Qiuting Wen, Jonathan Dietrich, Gregory M. Sokol, Senthilkumar Sadhasivam, Rupa Radhakrishnan

**Affiliations:** ^1^Department of Radiology and Imaging Sciences, Indiana University School of Medicine, Indianapolis, IN, United States; ^2^Department of Biostatistics and Health Data Science, Indiana University School of Medicine, Indianapolis, IN, United States; ^3^Indiana University School of Medicine, Indianapolis, IN, United States; ^4^Department of Pediatrics, Indiana University School of Medicine, Indianapolis, IN, United States; ^5^Department of Anesthesiology and Perioperative Medicine, University of Pittsburgh Medical Center, University of Pittsburgh School of Medicine, Pittsburgh, PA, United States

**Keywords:** NOWS, DTI, diffusion tensor imaging, brain development, prenatal opioid exposure, brain networks, opioid use disorder, structural connectivity

## Abstract

**Introduction:**

Infants with prenatal opioid exposure (POE) are shown to be at risk for poor long-term neurobehavioral and cognitive outcomes. Early detection of brain developmental alterations on neuroimaging could help in understanding the effect of opioids on the developing brain. Recent studies have shown altered brain functional network connectivity through the application of graph theoretical modeling, in infants with POE. In this study, we assess global brain structural connectivity through diffusion tensor imaging (DTI) metrics and apply graph theoretical modeling to brain structural connectivity in infants with POE.

**Methods:**

In this prospective observational study in infants with POE and control infants, brain MRI including DTI was performed before completion of 3 months corrected postmenstrual age. Tractography was performed on the whole brain using a deterministic fiber tracking algorithm. Pairwise connectivity and network measure were calculated based on fiber count and fractional anisotropy (FA) values. Graph theoretical metrics were also derived.

**Results:**

There were 11 POE and 18 unexposed infants included in the analysis. Pairwise connectivity based on fiber count showed alterations in 32 connections. Pairwise connectivity based on FA values showed alterations in 24 connections. Connections between the right superior frontal gyrus and right paracentral lobule and between the right superior occipital gyrus and right fusiform gyrus were significantly different after adjusting for multiple comparisons between POE infants and unexposed controls. Additionally, alterations in graph theoretical network metrics were identified with fiber count and FA value derived tracts.

**Conclusion:**

Comparisons show significant differences in fiber count in two structural connections. The long-term clinical outcomes related to these findings may be assessed in longitudinal follow-up studies.

## Introduction

Over 6% of pregnant women in the US indicate antepartum opioid use (Ko et al., 2020). From 1999 to 2014, opioid use in pregnant women increased more than 4-fold, (Haight et al., 2018) with consequent increase in incidence of infants born with prenatal opioid exposure (POE). Up to 94% of infants with POE develop drug withdrawal symptoms called neonatal opioid withdrawal syndrome (NOWS) (Khan, 2020). From 2004 to 2014, documented cases of NOWS rose 5-fold and NOWS management associated Medicaid costs rose from $65 million to $462 million, a greater than 7-fold increase (Winkelman et al., 2018).

Risks associated with POE go beyond signs and symptoms of NOWS. Children with POE are at a higher risk of adverse neurodevelopmental and neurobehavioral outcomes, such as learning and attention problems (Levine and Woodward, 2018), educational delay (Lee et al., 2019), and lower cognitive and motor scores than unexposed children (Yeoh et al., 2019). In addition, children with POE are at a higher risk for development of attention deficit hyperactive disorder (ADHD) (Azuine et al., 2019; Schwartz et al., 2021) and autism spectrum disorder (Rubenstein et al., 2019).

Older children and adolescents with a history of POE may have long-term alterations in brain development (Sirnes et al., 2017) compared to unexposed controls, including reduced basal ganglia, thalamus, and cerebellar white matter volumes (Walhovd et al., 2007; Yuan et al., 2014), reduced regional cortical thickness (Nygaard et al., 2018), and alterations in hippocampal volumes (Riggins et al., 2012; Robey et al., 2014). However, some of these alterations, such as cortical thickness, are not consistently identified in all studies (Walhovd et al., 2015; Sirnes et al., 2017) or the changes were negated by early childhood environment, Riggins et al. (2012) suggesting that perinatal and postnatal factors could potentially influence neuroplasticity in the developing pediatric brain. Older children and adolescents with POE also showed white matter microstructural alterations (Soares et al., 2013) alterations such as decreased fractional anisotropy (FA) (Walhovd et al., 2010) on diffusion tensor imaging (DTI), and alterations in working memory networks on functional MRI (fMRI) (Sirnes et al., 2018), all of which correlated with cognitive outcomes.

Recent studies have focused on early detection of evidence related to the impact of prenatal opioids on fetal and infant brain development. For example, fetal brains with POE demonstrated differences in brain morphometry (Radhakrishnan et al., 2022a) compared to unexposed controls based on fetal MRI. Alterations in functional network connectivity on resting state functional MRI (rs-fMRI) have also been described in infants with POE compared to controls using both seed-based and connectome-based approaches (Radhakrishnan et al., 2020, 2022b,c; Merhar et al., 2021; Liu et al., 2022). In a few small DTI studies, white matter microstructural alterations in infants with POE and substance exposure have been reported with voxel-wise analysis (Walhovd et al., 2012; Monnelly et al., 2018; Warton et al., 2018). In our study, we attempt to use a connectome-based approach to understand tract-based microstructural alterations in prenatal opioid exposure.

Brain connectivity is based on anatomical links (structural connectivity) and statistical dependencies (functional connectivity) (Rubinov and Sporns, 2010). It has been effectivity utilized to assess infant (Wen X. et al., 2019; Zhao et al., 2019) and even fetal brains (Turk et al., 2019). In infants, DTI has been used as a marker of white matter integrity and maturation in addition to POE, is shown to be altered in several conditions such as prematurity (Rose et al., 2014; Li et al., 2015; Pannek et al., 2018) and perinatal brain injury (Merhar et al., 2016; Kline-Fath et al., 2018). On rs-fMRI studies, the developing neonatal and infant brain shows increasing complexity in connectomes when assessed using graph theoretical models, with disruptions in network connectivity shown to be associated with perinatal brain insults (Zhao et al., 2019). Graph theory analysis involves studying relationships/connections mathematically utilizing systems composed of nodes and edges (connections between nodes). While the first paper involving graph theoretical analysis was first published in the 1736 by Swiss mathematician Leonhard Euler (Biggs et al., 1986), graph theory metrics have been applied in neuroimaging to understand networks in the human brain (Bassett and Bullmore, 2009; Bullmore and Sporns, 2009; Mears and Pollard, 2016; Sporns, 2018; Lee et al., 2020).

The direct effects of opioids on the developing brain are mainly through the opioid receptors which are expressed in variable concentrations in different regions of the developing brain as revealed in animal studies (Zhu et al., 1998). Opioid receptors are expressed on oligodendrocytes and their precursors as well as developing neurons. Specifically, these animal studies suggest that opioids impair regional brain myelination, probably through accelerated apoptosis of oligodendrocytes and microglial activation (Oberoi et al., 2019; Gibson et al., 2022). Studies also reveal increases in myelinated axons with compacted myelin sheaths (Vestal-Laborde et al., 2014) in prenatal methadone exposed rats, and dose dependent decreased number of myelinated axons and increased percentage of larger diameter axons with thinner myelin sheaths in prenatal buprenorphine exposed rats, with some of these changes being dose dependent (Sanchez et al., 2008). In addition, prenatal morphine exposure in rats have shown to be associated with regional decreases in neuronal dendritic length and branching (Mei et al., 2009). More recently, POE has been linked to increased neuroinflammation, reduced myelin basic protein, lower fractional anisotropy, and deficits in learning and executive control (Jantzie et al., 2020). These studies suggest an impact of prenatal opioid exposure on white matter developmental integrity that could be evaluated in the infant brain using DTI metrics. We therefore used a connectomics approach to assess microstructural brain development in infants with POE compared to control non-opioid exposed controls.

## Materials and methods

### Subject recruitment

Subject recruitment was performed similarly to previous studies (Radhakrishnan et al., 2022b,c). This prospective study was performed at Indiana University Health with approval by the Indiana University Institutional Review Board. We recruited infants with prenatal opioid exposure as well as control infants without prenatal opioid exposure at less than 3 months corrected postmenstrual age. Eligible participants were screened from medical records. Infants with major genetic or congenital anomalies, or significant postnatal abnormalities such as birth asphyxia or neonatal sepsis were excluded. Information regarding maternal and infant demographics, maternal opioid use, infant birth and postnatal details including any treatment for neonatal opioid withdrawal syndrome were collected from medical records and maternal self-report questionnaires. Written informed consent was obtained from at least one parent for all minor participants.

### Diffusion tensor imaging acquisition

MRI data were acquired on a single Siemens Prisma 3T scanner with a 64-channel RF receiver head/neck coil. All participants underwent T1-weighted imaging and diffusion MRI. T1-weighted anatomical imaging used a 3-dimensional magnetization rapid gradient echo (MPRAGE) with 1 mm × 1 mm × 1mm resolution. The diffusion MRI protocol employed a single-shot spin-echo echo-planar imaging (SS-SE-EPI) sequence with two diffusion-encoding schemes. One used a hybrid diffusion imaging (HYDI)-encoding scheme that contained three zero diffusion-weighting (i.e., *b*-value = 0 s/mm^2^) and multiple concentric diffusion-weighting shells (*b*-values = 5, 495, 500, 505, 795, 800, 805, 1590, 1595, 1600, 1605, 1610, 2590, 2595, 2600, 2605, and 2610 s/mm^2^) for a total of 141 diffusion-weighting gradient directions (Wu and Alexander, 2007; Wen Q. et al., 2019). The second scheme used a single shell diffusion imaging with 64 diffusion directions at *b*-value = 1000 s/mm^2^. The resolution was matched in both schemes, with a field of view of 160 × 160 mm, 66 slices, and a slice thickness of 1.5 mm, yielding 1.5-mm isotropic voxels. An additional *b* = 0 s/mm^2^ with reversed-phase encoding was acquired for geometric distortion correction.

### Preprocessing

Initial preprocessing of the MR images for each subject was performed using the FMRIB (for Functional MRI of the Brain) software Library (FSL, Oxford, UK) (Woolrich et al., 2009). Fieldmap and gradient-non-linearity distortion corrections were performed using FSL-topup (Smith et al., 2004). Eddy current-induced disruptions and subject motion were corrected using FSL-eddy (Andersson and Sotiropoulos, 2016). Samples were inspected for quality and those with deformations or greater than 20% loss were excluded. The b-tables were imported and corrected using DSI Studio^1^ using a population average template (Yeh et al., 2018). Diffusion data was reconstructed in the MNI space using q-space diffeomorphic reconstruction (QSDR) (Yeh and Tseng, 2011) and aligned with the software neonatal template. We note that for the HYDI-encoding scheme, only the *b*-values between 505 and 800 s/mm^2^ were included in the subsequent tractography analysis.

### Tractography

Tractography was performed on the whole brain with DSI Studio using a deterministic fiber tracking algorithm (Yeh et al., 2013) with a diffusion sampling ratio of 1.25. Ten million tracts were calculated for each subject. Quantitative anisotropy (qa) threshold values were determined based visual inspection to maximize number of tracts while minimizing spurious fibers. The quantitative anisotropy threshold was set at 0.05 and 0.03 for DTI and HYDI samples, respectively. The angular threshold was set at 45 degrees and the step size was 0.75 mm. Track lengths shorter than 10 mm or longer than 150 mm were discarded. ICBM template (Fonov et al., 2009, 2011) was registered to subject space through non-linear transformation. Brain parcelation regions were based on the AAL2 atlas (Rolls et al., 2015). Connectivity matrices and graph network measures were calculated in DSI Studio based on fiber count and fractional anisotropy (FA) values.

### Pairwise connectivity analysis

Connectivity matrices with 120 regions of interest (ROIs) were collected for each subject based on fiber count and FA values. Regions involving the cerebellum and vermis were excluded, leaving 94 ROIs for the analysis. Region pairs with zero values in over 50% of the samples were removed from the analysis ending up with 644 pairs of fiber count and 652 pairs of FA values. Linear regression models were fitted for each pair to compare between POE and control groups with infant sex, postmenstrual age (PMA) at time of MRI, and DTI scan sequence as the covariates. Multiplicity was corrected following the Benjamini-Hochberg procedure to control for the false discovery rate (Benjamini and Hochberg, 1995). A *p*-value of <0.05 was considered significant.

### Network measures

Network measures for fiber count and FA values were collected for each subject within DSI Studio using the Brain Connectivity Toolbox (Rubinov and Sporns, 2010). Evaluated network measures include network density, global clustering coefficients, local clustering coefficients, average path length, global efficiency, local efficiency, eccentricity, rich club coefficients, node degree, node strength, betweenness centrality, eigenvector centrality, and pagerank centrality. Network measures were based on weighted values. The same type of analysis as in the pairwise connectivity analysis was conducted with the network measures as the outcome. Multiple testing correction was also performed following the Benjamini-Hochberg procedure. A *p*-value of <0.05 was considered significant.

## Results

### Demographics

There were 37 subjects with DTI available and 8 infants were excluded due to poor DTI quality. Therefore, 29 infants were included in this study including 11 POE and 18 unexposed infants. Of these, 2 POE and 4 unexposed subjects were imaged using the HYDI protocol. The POE group demonstrated significantly lower birth weight than controls although mean birth weight for both groups was still within range of normal. There were no significant differences in gestational age at birth, postmenstrual age at time of MRI, or birth head circumference. There were differences in education levels as 6 of 18 mothers of unexposed infants had a college degree while none of the 11 mothers with POE infants were college graduates. Within the control group, there were no statistically significant differences in birth weight or birth head circumference in infants born to mothers with or without a college degree.

Mothers of the POE infant group received treatment with 10 receiving buprenorphine and 1 receiving methadone. Of the mothers with POE infants, 3 indicated use of opioids including heroin and 5 indicated use of other non-opioid substances including methamphetamines, marijuana, and cocaine. Demographic information for the 29 infants is shown in Table 1.

**TABLE 1:** Demographic information of subjects.

	POE	Control	*p*-value
Subject number	11	18	
Males	5	9	1
Gestational age (weeks) (SD)	37.71 (2.77)	39.59 (0.8)	0.051
Postmenstrual age at MRI (weeks) (SD)	43.42 (3.28)	44.37 (1.86)	0.4
Birth weight (kg) (SD)	2.715 (0.52)	3.386 (0.4)	0.002
Birth head circumference (cm) (SD)	33.16 (2.0)	34.39 (1.04)	0.083
Maternal age (years) (SD)	28.73 (3.56)	28.39 (4.27)	0.82
NOWS	3	NA	NA
Postnatal opioid treatment	2	NA	NA
Maternal depression	5	2	0.071
Maternal hepatitis	2	0	0.14
Maternal college degree	0	6	0.058
Maternal buprenorphine	10	NA	NA
Maternal methadone	1	NA	NA
Maternal other opioids	3	NA	NA
Maternal non-opioids	5	NA	NA
Maternal smoking	6	1	0.006

P-values were calculated using Fisher’s exact test or unpaired t-test as appropriate.

### Pairwise connectivity based on fiber count

**FIGURE 1:**
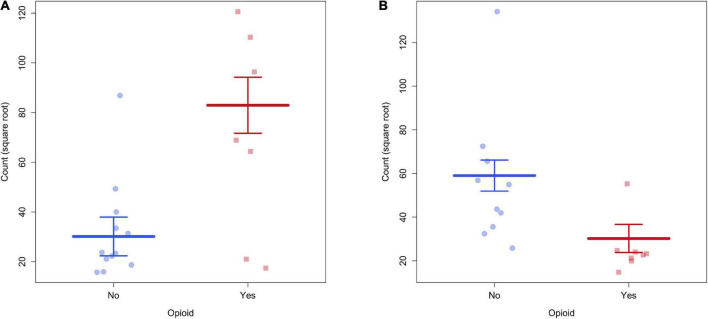
**(A)** There was greater structural connectivity between the right superior frontal gyrus and right paracentral lobule in POE infants. **(B)** There was greater structural connectivity between right superior occipital gyrus and right fusiform gyrus in unexposed infants.

**FIGURE 2:**
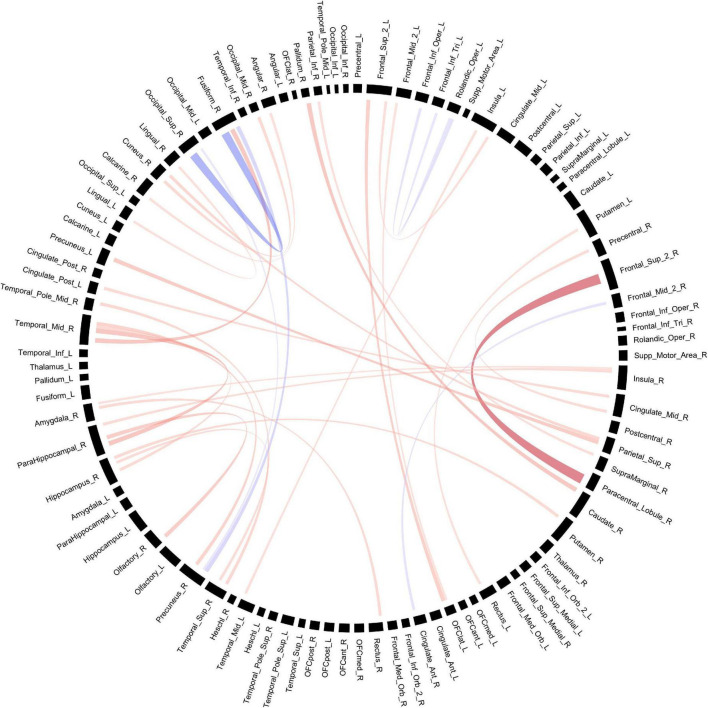
Structural connectivity between regions of interest based on fiber count. Red bands indicate a greater connectivity in the POE group while blue bands indicate greater connectivity in the control group. Band thickness is determined by the t-stat value.

Connectivity based on fiber count between multiple ROIs were significantly altered in POE infants compared to opioid naïve controls. Connections between the right superior frontal gyrus and right paracentral lobule and between the right superior occipital gyrus and right fusiform gyrus showed significance considering uncorrected *p*-values as well as p-FDR values (Figure 1). There were 30 other connections which were significant when considering uncorrected *p*-values but did not maintain significance when correcting for multiple comparisons. Diagram of connections based on fiber count is shown in Figure 2. Data is summarized in Table 2.

**TABLE 2:** Connectivity values between POE and opioid naïve infants based on fiber count.

ROI 1	ROI 2	POE vs. Cont. (SE)	t-stat	*p*-value	*p*-corr
Superior Frontal - R	Paracentral Lobule - R	52.80 (6.02)	8.77	4.60E−07	2.96E−04
Superior Occipital - R	Fusiform - R	−28.81 (4.08)	−7.06	8.53E−06	0.003
Parahippocampal – R	Middle Temporal - R	79.13 (19.38)	4.08	5.33E−04	0.092
Fusiform - R	Middle Temporal - R	83.61 (21.06)	3.97	5.68E−04	0.092
Caudate - R	Pallidum - R	54.85 (14.13)	3.88	7.12E−04	0.092
Superior Parietal - R	Precuneus - L	63.83 (17.53)	3.64	0.003	0.263
Olfactory - R	Parahippocampal – R	37.22 (10.79)	3.45	0.003	0.263
Fusiform - R	Precuneus - R	−43.12 (12.16)	−3.55	0.005	0.427
Superior Frontal - L	Lateral OFC - L	35.05 (10.5)	3.34	0.007	0.453
Precuneus - R	Middle Temporal - R	53.15 (17.2)	3.09	0.007	0.453
Superior Temporal - R	Middle Temporal Pole - R	18.97421	2.79	0.012	0.631
Cuneus - R	Superior Parietal - R	51.08212	2.74	0.012	0.631
Cuneus - R	Middle Occipital - R	52.65 (19.63)	2.68	0.013	0.631
Hippocampus - R	Putamen - R	33.87 (12.69)	2.67	0.014	0.631
Hippocampus - R	Middle Temporal - R	82.98 (31.96)	2.60	0.016	0.677
Middle Cingulate - R	Posterior Cingulate - L	34.47 (13.27)	2.60	0.017	0.677
Inferior Frontal (Oper) - L	Rolandic Operculum - L	−31.07 (12.32)	−2.52	0.019	0.709
Precentral - R	Middle Cingulate - R	47.57 (19.07)	2.49	0.021	0.750
Rectus - R	Amygdala - R	27.67 (10.54)	2.63	0.024	0.750
Insula - R	Parahippocampal - R	30.89 (12.37)	2.50	0.024	0.750
Hippocampus - R	Superior Temporal - R	46.81 (19.26)	2.43	0.025	0.750
Middle Frontal - R	Anterior Cingulate - R	−51.87 (21.32)	−2.43	0.026	0.750
Inferior Parietal - R	Supramarginal - R	30.14 (12.98)	2.32	0.029	0.814
Superior Frontal 2 - L	Insula - L	71.08 (30.49)	2.33	0.032	0.847
Insula - L	Middle Temporal - L	45.74 (20.46)	2.24	0.036	0.901
Rectus - L	Putamen - L	26.11 (11.69)	2.23	0.038	0.901
Inferior Frontal (Tri) - L	Rolandic Operculum - L	−35.88 (15.84)	−2.26	0.038	0.901
Calcarine - R	Angular - R	28.17 (12.69)	2.22	0.042	0.931
Lingual - L	Lingual - R	66.69 (30.76)	2.17	0.048	0.931
Insula - R	Amygdala - R	35.53 (16.73)	2.12	0.049	0.931
Middle Frontal - L	Lateral OFC - L	26.36 (12.47)	2.11	0.049	0.931
Superior Occipital - R	Precuneus - R	−52.34 (25.25)	−2.07	0.049	0.931

### Pairwise connectivity based on fractional anisotropy

**FIGURE 3:**
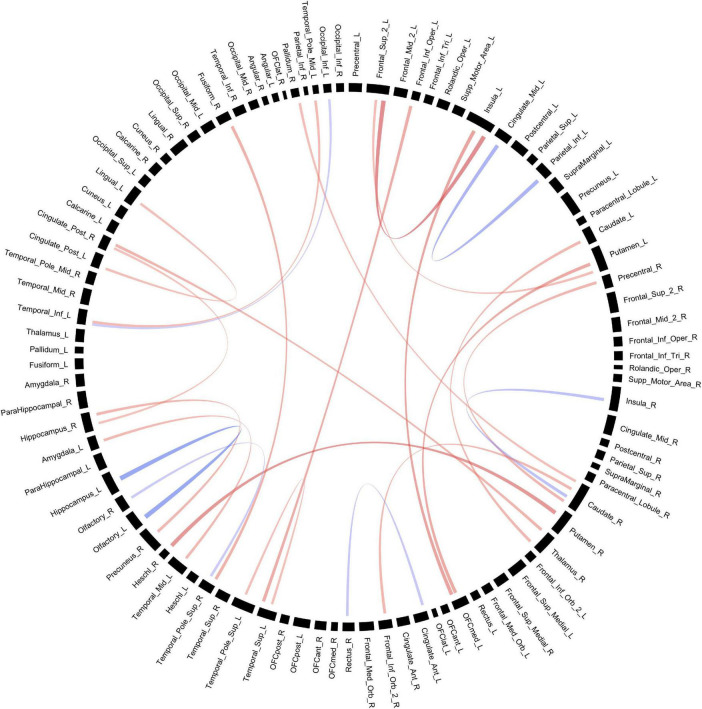
Structural connectivity between regions of interest based on FA values. Red bands indicate a greater connectivity in the POE group while blue bands indicate greater connectivity in the control group. Band thickness is determined by the t-stat value.

Connectivity based on FA between multiple ROIs were altered in POE infants compared to opioid naïve controls. Prior to multiple correction, there were 24 connections which were significant when considering uncorrected *p*-values, but none of these retained significance after correcting for multiple comparisons. Diagram of connections based on FA values is shown in Figure 3. Data is summarized in Table 3.

**TABLE 3:** Connectivity values between POE and opioid naïve infants based on fractional anisotropy (FA).

ROI 1	ROI 2	POE vs. Cont. (SE)	t-stat	*p*-value	*p*-corr
Superior Frontal – L	Insula - L	0.041 (0.011)	3.78	0.001	0.47
Olfactory – L	Hippocampus – L	−0.044 (0.011)	−3.96	0.001	0.47
Putamen – R	Heschl’s- R	0.044 (0.013)	3.46	0.003	0.65
Middle Cingulate – L	Inferior Parietal - L	−0.026 (0.008)	−3.31	0.006	0.98
Medial OFC – L	Insula - L	0.04 (0.013)	3.07	0.010	0.98
Insula - R	Caudate - R	−0.041 (0.015)	-2.66	0.014	0.98
Inferior Frontal (Oper) – L	Superior Temporal - L	0.059 (0.021)	2.76	0.016	0.98
Medial OFC - L	Putamen - L	0.029 (0.01)	2.83	0.016	0.98
Fusiform - R	Superior Temporal Pole - R	0.036 (0.014)	2.62	0.021	0.98
Posterior Cingulate - R	Thalamus - R	0.082 (0.033)	2.50	0.023	0.98
Hippocampus - R	Precuneus - R	0.034 (0.014)	2.42	0.024	0.98
Inferior Frontal (Orb) – R	Caudate - R	0.032 (0.013)	2.42	0.029	0.98
Caudate – L	Thalamus - R	0.065 (0.027)	2.42	0.034	0.98
Middle Temporal_Pole - L	Inferior Temporal - L	0.018 (0.008)	2.28	0.034	0.98
Precentral - R	Caudate - R	0.038 (0.015)	2.45	0.034	0.98
Amygdala - L	Middle Temporal - L	0.031 (0.013)	2.33	0.035	0.98
Rectus - R	Anterior Cingulate - L	−0.071 (0.03)	−2.34	0.036	0.98
Caudate - R	Palladium - R	0.032 (0.015)	2.18	0.039	0.98
Olfactory - R	Superior Temporal Pole - R	−0.03 (0.013)	−2.24	0.039	0.98
Superior Frontal – L	Putamen - L	0.023 (0.011)	2.18	0.044	0.98
Posterior Cingulate - L	Lingual - L	0.043 (0.02)	2.15	0.046	0.98
Superior Temporal - L	Superior Temporal Pole - L	0.023 (0.011)	2.10	0.046	0.98
Inferior Occipital - L	Inferior Temporal - L	−0.021 (0.01)	−2.11	0.046	0.98
Posterior Cingulate - R	Hippocampus - R	0.05 (0.024)	2.06	0.050	0.98

### Network measures based on fiber count

Based on fiber count, there were 15 network metrics which were significantly altered in POE infants compared to opioid naïve controls when considering uncorrected *p*-values. None of the networks maintained statistical significance when correcting for multiple comparisons. Data is summarized in Table 4.

**TABLE 4:** Network measures between POE and opioid naïve infants based on fiber count.

Network Measure	POE vs. Cont. (SE)	t-stat	*p*-value	*p*-corr
**Betweenness centrality**				
Transverse Temporal – L	10.52 (4.98)	2.11	0.045	0.694
Inferior Parietal – L	43.3 (19.65)	2.20	0.037	0.694
Supp. Motor – R	−44.66 (20.68)	−2.16	0.041	0.694
Thalamus – L	357.25 (121.32)	2.94	0.007	0.665
**Cluster coefficient**				
Caudate – R	0.005 (0.002)	2.43	0.023	0.691
Middle Cingulate – R	0.007 (0.002)	3.44	0.002	0.201
Hippocampus – R	0.01 (0.004)	2.43	0.023	0.691
Inferior Parietal – L	−0.013 (0.006)	−2.21	0.037	0.691
Middle Temporal – R	0.009 (0.004)	2.26	0.033	0.691
**Eigenvector centrality**				
Precentral – R	−0.025 (0.012)	−2.12	0.045	0.997
**Local efficiency**				
Caudate – R	0.009 (0.004)	2.43	0.023	0.724
Middle Cingulate – R	0.011 (0.004)	2.68	0.013	0.724
Hippocampus – R	0.018 (0.007)	2.58	0.016	0.724
Inferior Parietal – L	−0.019 (0.009)	−2.27	0.032	0.764
**Pagerank centrality**				
Precentral – R	−0.002 (0.001)	−2.48	0.020	0.771

### Network measures based on fractional anisotropy

Based on FA values, there were 22 networks which were significantly altered in POE infants compared to opioid naïve controls when considering uncorrected *p*-values. None of the networks maintained statistical significance when correcting for multiple comparisons. Data is summarized in Table 5.

**TABLE 5:** Network measures between POE and opioid naïve infants based on fractional anisotropy (FA).

Network Measure	POE vs. Cont. (SE)	t-stat	*p*-value	*p*-corr
**Betweenness centrality**				
Cuneus – R	93.3 (27.95)	3.34	0.003	0.086
Hippocampus – L	−194.23 (81.31)	−2.39	0.025	0.273
Hippocampus – R	−118.81 (42.54)	−2.79	0.010	0.19
Lateral OFC – L	−1.58 (0.74)	−2.13	0.043	0.369
Medial OFC – R	36.38 (15.59)	2.33	0.028	0.273
Inferior Parietal – L	65.37 (18.97)	3.45	0.002	0.086
Rectus – L	205.45 (75.78)	2.71	0.012	0.191
Rectus – R	168.55 (47.09)	3.58	0.002	0.086
Supp. Motor – R	−70.59 (29.11)	−2.43	0.023	0.273
Supramarginal – ‘R	47.63 (20.51)	2.32	0.029	0.273
Thalamus – R	−153.34 (54.79)	−2.80	0.010	0.19
**Cluster coefficient**				
Middle Occipital – L	−0.033 (0.014)	−2.28	0.032	0.925
Rectus – R	−0.042 (0.019)	−2.22	0.036	0.925
Supp. Motor – L	−0.027 (0.013)	−2.13	0.044	0.925
Thalamus – L	−0.04 (0.018)	−2.24	0.034	0.925
**Local efficiency**				
Middle Occipital – R	−0.048 (0.022)	−2.16	0.041	0.853
Paracentral Lobule – R	−0.052 (0.02)	−2.54	0.018	0.853
**Pagerank centrality**				
Middle Occipital – R	−0.001 (0.0006)	−2.08	0.049	0.761
Medial OFC – L	0.001 (0.0006)	2.22	0.036	0.761
Precuneus – L	−0.002 (0.0007)	−2.47	0.021	0.761
Supp. Motor – R	−0.002 (0.0006)	−3.15	0.004	0.408
Inferior Temporal – L	0.001 (0.0005)	2.12	0.044	0.761

## Discussion

Our study is one of the first to show alterations in structural ROI-to-ROI pairwise connectivity and structural network measures in infants with POE compared to non-opioid exposed control infants. In infants with POE, there were greater number of fiber tracks between the right superior frontal gyrus and the right paracentral lobule and fewer fiber tracks between the right superior occipital gyrus and right fusiform gyrus when compared to control infants. We also identified several other regional alterations in graph network metrics in infants with POE; however, these did not maintain statistical significance after multiple corrections.

A few small studies have investigated brain diffusion metrics in infants with prenatal opioid exposure (Walhovd et al., 2012; Monnelly et al., 2018; Peterson et al., 2020). Using tract based spatial statistics, Monnelly et al. showed decreased fractional anisotropy in the internal capsule and inferior longitudinal fasciculus in prenatal methadone exposed infants compared to controls (Monnelly et al., 2018). Using similar methods, Walhovd et al. identified higher mean diffusivity (MD) in the superior longitudinal fasciculus in methadone exposed infants compared to control infants (Walhovd et al., 2012). Interestingly, another recent study showed higher FA values and reduced apparent diffusion coefficient (ADC) values in the frontal and parietal white matter in infants with prenatal methadone or heroin exposure (Peterson et al., 2020). In our current study, we used graph theoretical modeling to better understand global brain structural connectivity. The apparent differences in direction of FA values on prior studies is somewhat explained by our study, where we identified both stronger and weaker ROI-to-ROI connectivity based on FA strength in different brain region pairs in infants with POE compared to controls. We hypothesize therefore that there may be regional alterations in myelin and axonal microstructure that could be responsible for these results. Opioids likely exert their effects on the brain mainly through the opioid receptors and animal studies show differential opioid receptor expression in the developing fetal brain (Zhu et al., 1998). Opioid receptors are expressed on neurons and oligodendrocytes and their precursors, and are responsible for neuronal and glial development, and prenatal exposure to exogenous opioids may hence result in regional variations in degree of neuronal apoptosis and myelination in the fetal and infant brain (Vestal-Laborde et al., 2014; Velasco et al., 2021).

We identified significantly higher FA based fiber tracts in the right superior frontal gyrus to the right paracentral lobule. The superior frontal gyrus has been associated with response inhibition and motor urgency (Hu et al., 2016), while the paracentral lobule is responsible for motor and sensory functions of the lower limbs (Johns, 2014). Cortical thickness in the right superior frontal gyrus and other areas of the right prefrontal cortex (Almeida et al., 2010) are also shown to be reduced in children and adults with attention deficit hyperactivity disorder (ADHD). Considering that those with POE are more likely to develop ADHD (Azuine et al., 2019; Schwartz et al., 2021), assessing structural connectivity of the superior frontal gyrus may be an important prognostic marker for developmental disorders such as ADHD.

We also identified a significant lower FA based fiber count between the right superior occipital gyrus and the right fusiform gyrus in infants with POE compared to controls. Both these regions correspond to visual processing and object and facial recognition (Weiner and Zilles, 2016). Our results are in keeping with other studies have also indicated visual deficits in children with POE (McGlone and Mactier, 2015; Maguire et al., 2016; Andersen et al., 2020). Children with POE have shown to have vision impairments compared to prenatally unexposed children (Andersen et al., 2020). Additionally, these children have been linked to poorer visual motor skills (Melinder et al., 2013; Sundelin-Wahlsten and Sarman, 2013).

Structural changes in brain functional connectivity have also been shown in infants with POE. Recent work showed alterations in amygdala and thalamocortical functional connectivity in infants with POE compared to controls (Radhakrishnan et al., 2020, 2022b). In addition, recent studies have also applied graph theoretical methods to understand further the brain functional connectivity alterations in POE and their associations with maternal comorbidities and clinical outcomes (Merhar et al., 2021; Radhakrishnan et al., 2022c). Due to differences in brain segmentation atlases, ROI selections and network connectivity analyses methods used in these prior rs-fMRI studies compared to our current DTI study, intermediary causal relationship of microstructural to functional network alterations is difficult to surmise. Since structural deficits often underlie functional impairments, we may presume some degree of overlap in regional structural and functional connectivity. However, prenatal opioid exposure is known impact myelin, axonal and neuronal integrity based on the distribution of opioid receptors, and there may also be isolated effects of opioids on these structures. Brain morphometry, DTI and fMRI network studies may therefore be considered complementary in assessing the overall impact of opioids on the developing brain.

Several mothers in our study indicated use of substances such as methamphetamines, marijuana, cocaine, and tobacco. Prenatal exposure to some of these substances also show disruptions in the structural connectome. For example, neonates with prenatal methamphetamine exposure (PME) show lower FA values than unexposed infants in several connections between the striatum and midbrain, orbitofrontal cortex and associated limbic structures, all components of the striato-thalamo-orbitofrontal circuit and its limbic connections, which may be responsible for drug addiction related neurocircuitry (Warton et al., 2018, 2020). Detrimental effects due to prenatal substance exposure may linger as older children with PME demonstrated lower FA in the frontal and limbic regions and greater mean, radial, and axial diffusivities (Roos et al., 2015) as well as lower apparent diffusivity coefficients (Cloak et al., 2009). These early brain microstructural alterations noted on diffusion imaging in prenatal cocaine, marijuana and methadone or heroin exposure may also serve as predictors of 12 month behavioral and language outcomes (Peterson et al., 2020). Similarly, infants with prenatal alcohol exposure also showed altered FA values and reduced white matter microstructural integrity (Donald et al., 2015), and these early brain microstructural disruptions may persist until later life (Wozniak et al., 2009; Moore and Xia, 2021; Roos et al., 2021; Stephen et al., 2021).

Given the long-term developmental issues associated with prenatal substance exposure, especially in the realm of mental processing, impulse control, and executive functions (Wozniak and Muetzel, 2011), identification of early brain developmental microstructural alterations with DTI may offer a mechanistic understanding and be a predictive biomarker for these future outcomes. However, more work needs to be done to further our understanding on how the brain structural connectome may correlate with developmental outcomes.

This study had a few limitations. First, this study was restricted by the number of subjects that were evaluated. While 37 infants were imaged, only 29 studies were able to be used for analysis due to poor image quality, mostly due to motion artifact. Since diffusion imaging has a low signal to noise ratio, it is particularly susceptible to motion artifacts (Farrell et al., 2007). Infants pose a particular challenge as they cannot follow instructions. While imaging of neonates in this study was conducted during sleep, head motion was still a noticeable issue. Two different diffusion techniques were used, however we had infants with POE and control subjects that were scanned with both these techniques, and DTI scan technique was used in the regression model to account for scan related issues. Polysubstance use was not controlled for in this study due to limited prevalence of individuals with single substance use. Polysubstance use (e.g., SSRIs, nicotine, benzodiazepines, etc.) is known to affect opioid metabolism and development of NOWS (Patrick et al., 2015) and may impact brain development. Future larger studies would be better powered to understand these associations. Maternal comorbidities (e.g., stress, depression, socioeconomic, genetic) were not included in this small sample study. We and other researchers have shown the effects of maternal comorbidities on infant brain functional connectivity, and we hope that future studies would be able to assess this impact. Nevertheless, our study has shown novel findings in graph theoretical networks of brain structural connectivity in POE that adds to the existing limited knowledge in this field. Future longitudinal studies can also help understand the clinical outcomes associated with brain microstructural alterations in prenatal substance exposure.

## Conclusion

Children with prenatal opioid exposure may be at greater risk to have a developmental disorder. Assessing structural connectivity could have important prognostic value for these conditions. This study identified two structural connections where tract counts were significantly different in POE infants compared to unexposed infants. These microstructural alterations may be positive or negative based on brain region and may reflect differences in development and opioid related impact. Future longitudinal studies with larger sample sizes would help understand how these preliminary results of altered structural connectivity relate to long-term developmental implications in children with prenatal substance exposure.

## Data availability statement

The raw data supporting the conclusions of this article will be made available by the authors, without undue reservation.

## Ethics statement

The studies involving human participants were reviewed and approved by the Indiana University Institutional Review Board. Written informed consent to participate in this study was provided by the participants’ legal guardian/next of kin.

## Author contributions

RV contributed to the sample processing, analysis, design, and manuscript preparation. YZ contributed to the analysis and manuscript preparation. QW contributed to the analysis, design, and manuscript preparation. JD contributed to the sample processing. GS and SS contributed to the recruitment and design. RR contributed to the recruitment, design, and manuscript preparation. All authors contributed to the article and approved the submitted version.

## Footnotes

1
http://dsi-studio.labsolver.org

## Abbreviations

R: right
L: left
POE: prenatal opioid exposure
NOWS: neonatal opioid withdrawal syndrome
ROI: region of interest
MRI: magnetic resonance imaging
DTI: diffusion tensor imaging
ADHD: attention deficit hyperactivity disorder.
